# Risk factors for Keshan disease: a prospective cohort study protocol of gut flora

**DOI:** 10.1186/s12872-020-01765-x

**Published:** 2020-11-11

**Authors:** Zhenzhen Li, Jin Wei, Yanping Zhang, Gaopeng Li, Huange Zhu, Na Lei, Qian He, Yan Geng, Jianhong Zhu

**Affiliations:** 1 Department of Clinical Laboratory, The Second Affiliated Hospital of Xi’an Jiaotong University, 157 Xiwu Road, Xi’an, 710004 People’s Republic of China; 2 Department of Cardiology, The Second Affiliated Hospital of Xi’an Jiaotong University, 157 Xiwu Road, Xi’an, 710004 People’s Republic of China; 3 Key Laboratory of Synthetic and Natural Functional Molecule Chemistry of the Ministry of Education, Shaanxi Key Laboratory of Physico-Inorganic Chemistry, College of Chemistry and Materials Science, Northwest University, Xi’an, 710127 People’s Republic of China; 4 Clinical Research Center for Endemic Disease of Shaanxi Provincerovince, 5 Jianqiang Road, Xi’an, 710004 People’s Republic of China

**Keywords:** Keshan disease, Gut flora, The big data platform, Cohort study, Metabonomic analysis

## Abstract

**Background:**

Keshan disease is an endemic cardiomyopathy of undefined causes. Being involved in the unclear pathogenesis of Keshan disease, a clear diagnosis, and effective treatment cannot be initiated. However, the rapid development of gut flora in cardiovascular disease combined with omics and big data platforms may promote the discovery of new diagnostic markers and provide new therapeutic options. This study aims to identify biomarkers for the early diagnosis and further explore new therapeutic targets for Keshan disease.

**Methods:**

This cohort study consists of two parts. Though the first part includes 300 participants, however, recruiting will be continued for the eligible participants. After rigorous screening, the blood samples, stools, electrocardiograms, and ultrasonic cardiogram data would be collected from participants to elucidate the relationship between gut flora and host. The second part includes a prospective follow-up study for every 6 months within 2 years. Finally, deep mining of big data and rapid machine learning will be employed to analyze the baseline data, experimental data, and clinical data to seek out the new biomarkers to predict the pathogenesis of Keshan disease.

**Discussion:**

Our study will clarify the distribution of gut flora in patients with Keshan disease and the abundance and population changes of gut flora in different stages of the disease. Through the big data platform analyze the relationship between environmental factors, clinical factors, and gut flora, the main factors affecting the occurrence of Keshan disease were identified, and the changed molecular pathways of gut flora were predicted. Finally, the specific gut flora and molecular pathways affecting Keshan disease were identified by metagenomics combined with metabonomic analysis.

*Trial registration*: ChiCTR1900026639. Registered on 16 October 2019.

## Background

Clinically, Keshan disease (KD) is characterized by myocardial injury and symptoms of cardiovascular diseases, such as heart failure and cardiac dysfunction [1]. Though it is prevalent in China for 80 years, however, the highest mortality and disability rate has been reported during the 1950s to the 1980s [2]. The most frequent occurrence area of KD is reported in Shaanxi province, where 29 districts including 236 villages of 6 cities are affected, with about more than 3.1 million population at risk [3, 4].

KD is an endemic cardiomyopathy with unknown etiology in China. After years of efforts, remarkable effects have been achieved in comprehensive prevention and control of KD, however, multifarious problems remain elusive [5] i.e., (1) The etiologies and pathogenesis of KD have not been fully elucidated, therefore, the hidden dangers of KD re-epidemic still exist [6]; (2) The detection rate of “dilated cardiomyopathy” (“DCM”) in KD-inflicted area is remarkably higher than that in non-disease area [3]. “DCM” showing cardiac enlargement might not be a valid dilated heart disease. Yet, it is unclear to differentiate DCM from KD [7] which impedes the development of effective and normative treatments of KD, particularly, chronic Keshan disease (CKD). CKD is a severe illness with high mortality and poor prognosis [8, 9]. However, the transformation mechanism of the latent Keshan disease (LKD) to the CKD remains unknown [10]. Hence, these above-mentioned issues regarding KD require urgent attention to be addressed.

International and domestic researchers have attempted to explore the pathophysiological process of KD from many different perspectives, such as environmental factors, nutrition, family epigenetic susceptibility, and so on [10–12]. Although up to now, the pathogenesis of KD remains unclear. Certainly, a treatment plan without a clear etiology may fail of remission and can lead to poor prognosis. Intriguingly, with in-depth research on cardiovascular diseases and intestinal microecology, the relationship between cardiovascular diseases and gut flora has been gradually recognized. Particularly the evidence that gut flora has affected the progress of cardiovascular diseases such as hypertension, coronary atherosclerosis, and heart failure will help researchers to predict the roots of KD. Considering the fact, we anticipate that gut flora may provide a new direction for the treatment of cardiovascular disease. Thus, KD is attributed to cardiovascular disease with complicated etiology and tends to be affected by environmental factors, lifestyles, diet habits, and genetic factors. Concurrently, these factors further are known to affect the distribution, function, and metabolism of gut flora as well [13, 14]. Intriguingly, since 2000, our team has been extensively studying the molecular genetics, gene polymorphism, gene methylation, and other changes of KD, as well as the effects of low selenium on myocardial mitochondrial proteomics and cardiac structure and function [12, 15–19]. In summary, we provide a pioneer and a comprehensive definite basis for the KD research.

Here, we established the KD baseline data database, biological sample bank, and clinical data sample bank. We attempted to set up this study from a novel perspective, commencing with the distribution and metabolic status of gut flora of KD. It will couple with proteomics, metabolomics, and metagenomics with final validation by implicating the big data analysis technology to dig deeply into the relationship between gut flora and KD. Hence, considering these above approaches, we are attempting to provide valuable contributions in the discovery of biomarkers and a comprehensive pathological mechanism of KD.

## Method/design

### Study aim

The purpose of this study is as follows: At first, we aim to elucidate the basic characteristics of gut flora in patients with KD and clarify the relationship between gut flora and the development of KD; The second part is designed to explore novel biomarkers for the diagnosis of KD; Finally, we determine that the combination of gut flora function analysis tools and the big data analysis platform. And most of all, the most likely etiology of KD can be predicted and analyzed, providing a new target for the treatment of KD.

### Study design

#### The initial phase

**Fig. 1:**
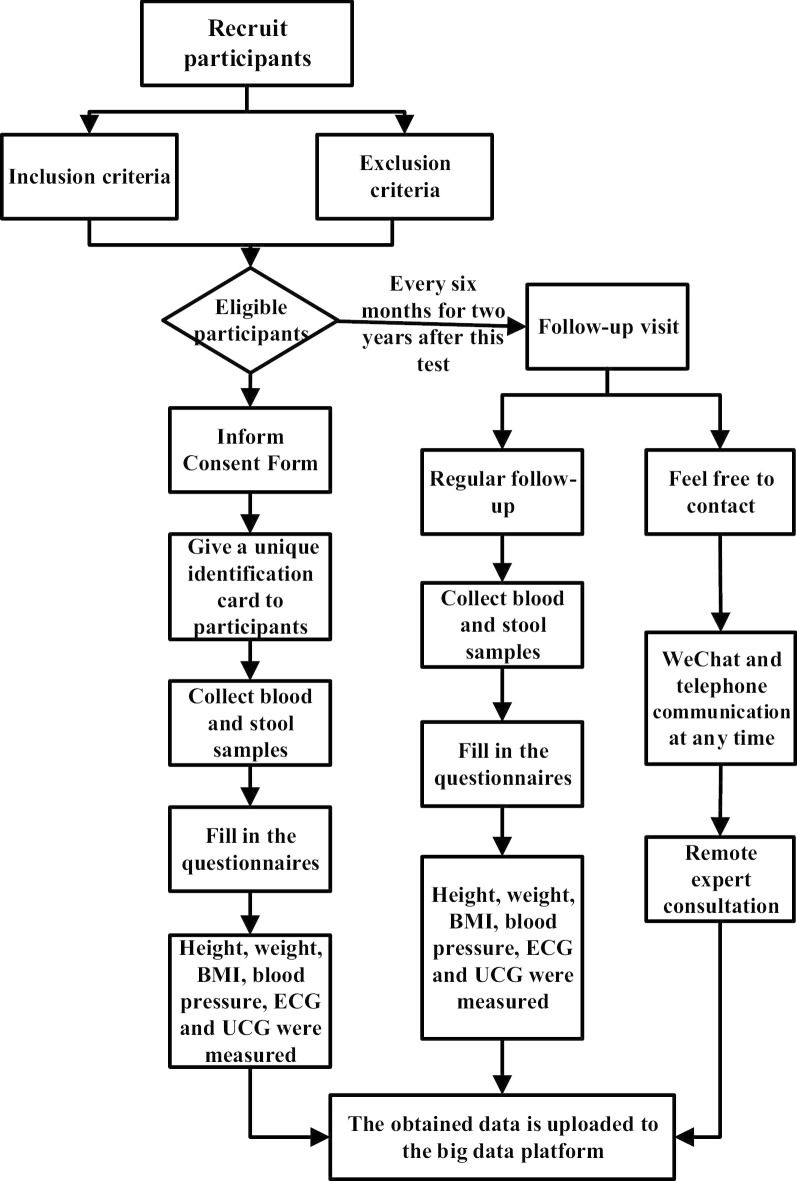
Flow chart of study design

In this part, our team consists of cardiologists, epidemiologists, pathologists, nutritionists, sports medicine doctors, geneticists, and nurses. Epidemiologists supervise the reliability of the entire study and will be responsible for regular monitoring of progress research (Fig. 1). Depending on the age distribution of patients, the next step will be conducted to screen out the suspected patients with KD, LKD, CMD, normal persons from wards, and DCM, normal persons from non-wards as our participants (Table 1), and then to conduct the systematic sampling with the 1:1 sex ratio.

**Table 1:** The source of the object of study

Disease-prone areas (case)				Non-ward areas (case)	
Suspected KD (50)	LKD (50)	CKD (50)	Normal ward control (50)	DCM (50)	Normal non-ward control (50)

Moreover, as part of our study design, in-ward and non-ward consultations will be made every 6 months in 2 years to collect baseline data and clinical samples of participants, simultaneously. Whereafter, these data will be uploaded to the confidential database along with the application of a remote diagnosis and treatment system for daily consultation and follow-up of participants.

#### The follow-up phase

The follow-up events will be conducted by a cardiologist and a nurse (Fig. 1). There will be two types of follow-up, one is a fixed free clinic twice a year, which will be carried out in detail according to the initial stage operation process; the other will be to ask the participant’s recent physical status by phone or WeChat every month (Fig. 1). All the data of follow-up and return visits will be accurately uploaded to the big data platform in a timely and accurate manner. The results will be evaluated and confirmed by three physicians. When the participants will be back in the clinic on schedule, we will inform the patients and interpret the results of the examination and the physician’s assessment reports. However, if the participants will not be able to come to the center due to unavoidable factors, the test report will be sent to them by mail and over the phone consultation by a cardiologist.

### Study objects

This study will recruit 300 participants among which 200 participants will be from Keshan disease-prone areas in Shaanxi Province and the other 100 participants were from five non-ward areas through a random consultation. This cohort will include the suspected KD (50 cases), LKD (50 cases), CKD (50 cases), normal ward control (50 cases), DCM (50 cases), and normal non-ward control (50 cases) (Table 1). According to our estimation, the final sample size could reach about 300 participants. The recruitment will start from January 1, 2020, to December 31, 2020, and the participants with satisfying eligibility criteria will be included in our study.

### Eligibility criteria

#### Inclusion criteria

1. Participants should be between the age of 18 and 70 years old.
2. The participant should have lived in the current place for at least 1 year and have no immigration plan for the next few years.
3. Suspected KD and KD patients will be preliminarily diagnosed and grouped by cardiovascular physicians according to the clinical diagnostic criteria of KD (WS/T 210–2011) and the severity of the disease.
4. Patients with DCM after initial diagnosis will be divided into groups by cardiovascular physicians according to the clinical diagnostic criteria of DCM.

#### Exclusion criteria

1. Participants having any mental or physical disability.
2. Participants having a history of coronary artery bypass, percutaneous coronary intervention, and positive angiography report.
3. Participants with diabetes, hypertension, thyroid disease, gastrointestinal diseases, major organ injury, mental illness, tumors, and infectious diseases.
4. Pregnant or lactating women.
5. Participants with a history of using any type of antibiotics or probiotics within 1 month.
6. The participants who will refuse to continue will be excluded.

### Outcomes

#### Minor outcome measures

The minor outcome will include healthy people or suspected KD develop into KD, and LKD develops into CKD.

#### Major outcome measures

The main outcomes will include acute Keshan disease attack, severe heart failure, perioperative period, and death.

### Recruitment

The participants will be informed of the purpose, content, risks, and benefits of this study. If they agree to participate, they should sign an informed consent form and would receive a schedule of this study (Fig. 1). The Clinical Research Center for Endemic Disease of Shaanxi province will review the reported information of participants from time to time via a big data network platform to ensure the orderly arrangement of experimental data.

### Study visit

#### The first part

- *Step 1* Collection of baseline data

Blood and stool samples will be obtained from participants to ensure whether they conform to the fasting instructions on the day of the program. Whereafter, the selected participants should sign an informed consent form. The organizer will then assign each participant with a special digital identification barcode. This bar code will be the unique identification of the participants and will accompany them throughout the entire study. However, this bar code will not mention the grouping thus participants.

- *Step 2* Sample collection

The blood samples will be collected in duplicate (Table 2). One tube of 5 ml (without anticoagulant) will be used to separate the serum for biochemical and mass spectrometry. Whereas the other tube of 2 ml blood (plus EDTA-2k anticoagulant) will be used to separate the plasma for brain natriuretic peptide (BNP) testing. Aliquoted serum and plasma should be stored at − 80 °C.

**Table 2:** Data collection using different methods at baseline

Questionnaires	Instruments	Blood sample	Stool
General questionnaire (in 10 sections)	ECG (electrocardiograph and heart rate) UCG (ultrasonic cardiogram) Physical activity questionnaire (IPAQ) Sphygmomanometer (blood pressure)	Hematological parameters (CBC)	Gut flora
Physical activity questionnaire (IPAQ)	Waist, hip, and wrist circumference	Biochemical parameters (total cholesterol, HDL cholesterol, LDL)	
Psychological status questionnaire			
Nutritional habits questionnaire (FFQ based on domestic foods)	Height scale and digital scale for weight	DNA extraction	

*HDL* high-density lipoprotein cholesterol, *LDL* low-density lipoprotein cholesterol

Stool (about 20 g) samples will be collected into an airtight container by the subjects themselves and then divided by the nurse and stored at − 80 °C for mass spectrometry and intestinal microflora analysis (Table 2).

- *Step 3* A questionnaire survey

After breakfast, participants will fill out a general questionnaire, physical activity questionnaire (IPAQ), psychological status questionnaire, and nutritional habits questionnaire (FFQ based on domestic foods) (Table 2).

- *Step 4* Functional testing

The parameters including index height, weight, wrist, waist, hip circumference, blood pressure, electrocardiograph (ECG), and heart rate, and ultrasonic cardiogram (UCG) functions will be obtained in this step (Table 2).

A professional physician will evaluate the above results. However, it will be further reviewed by a cardiologist to confirm the diagnosis and to recommend any necessary suggestions or requests to repeat the procedure.

#### The second part

After accomplishing the first phase, the participants will be informed by the test results or any possible warnings to require a higher medical consultation or other prescription. This free health check and a higher medical consultation will be provided with the motivation to start and continue the research.

### Data management and analysis

A research assistant will review the integrity and quality of the data to upload to the big data platform. The uploaded data will be further reviewed by the epidemiologists of the Clinical Research Center for Endemic Disease of Shaanxi province. Whilst unqualified data will be reacquired. Of note, the data output will be checked weekly.

Simultaneously, all the data will be uploaded to the official website of the Chinese Clinical Trial Registration Center for review. Based on previous findings and recommendations of the Health Services Standards Research Committee, baseline data and hybrid models will be analyzed in different categories and time-to-event analyses will be performed on subsequent data. Multiple inputs will be applied to deal with missing values.

## Limitations

- The fact that KD has a complicated etiology, therefore, it is difficult to distinguish its clinical symptoms from those of DCM.
- Limitations of this study include selection bias, as it includes only 18 to 70-year-olds and only the Chinese population, which may lead to the need to confirm the results of multinational studies.
- Of note, presently we do not know how many participants will continue to participate or leave this study, due to various reasons i.e., relocation, refusal to join, and other diseases and deaths. If there is a loss, we will immediately find the cause and remedy it. If it cannot be remedied, we will follow the last-observation-carried-forward (LOCF) method. Moreover, we will follow up with the participants to ensure the continuity of the data.

## Discussion

Despite having ample amounts of clinical cohorts of cardiovascular diseases in China, few gut flora studies have been focused on KD. The innovation of the present study lies in the combination of different methodologies which aim at collecting objective data concerning the characterization of the gut flora in KD patients, to highlight the variation in gut flora or mutual development of KD (normal ward, suspected KD, LKD, CKD), and to clarify the gut flora diversity between KD and DCM. Moreover, metagenomics metabolomics and metagenomics data will allow us to collect detailed information about the interaction between gut flora and KD along with the functional changes of gut flora in KD patients. Additionally, the inclusion of research tools of gut flora function and big data technology allow us to screen out novel biomarkers for early diagnosis and the molecular changes in the development of KD. Thus, it will provide a complete guideline to a novel perspective for the clinical treatment of KD. By looking at the defined causes of KD, we will be able to assess huge economic and social benefits for our country, by enhancing the chances of early prevention of KD, improving the prognosis of patients with KD, and even laying the foundation for the ultimate elimination of KD.

Though in recent years, many researchers have conducted extensive researches on the effects of genetic variation, gut flora, and its metabolites on cardiovascular diseases [20–23]. However, in the present study, it has been suggested that some people may have more than two risk factors, which attracts more researchers to search for common causes. The prospective study allows us to find the causes of KD from selenium deficiency, viral infection, nutritional deficiency, and other incentives. The fact our previously reported study could only determine a few aspects, however, here we attempted to identify the major cause. Furthermore, the implication of the perspective of genetics allows assessing that KD has certain heredities. Therefore, as a further strategy to determine the etiology of KD, we plan to establish a biological sample library to collect adequate samples and accumulate a large amount of useful data to fully prepare for later research.

Additionally, our team will invite each of the small and medium-sized hospitals in Shaanxi province to share their experience at the Clinical Research Center for Endemic Disease of Shaanxi province**,** to conduct a panel discussion of KD in every quarter. Moreover, we will regularly arrange the KD special lectures in the areas with high incidences to standardize the training of clinical centers in each hospital. Our team is very keen to help small and medium-sized hospitals to standardize the diagnosis and treatment of KD through the above-mentioned methods. In conclusion, through its multidisciplinary approach, the KD prospective cohort study allows the evaluation of risk factors for the development of KD and will also improve our understanding of the underlying pathogenic mechanism. Nevertheless, we also aim to spread the knowledge of KD to the patients to prevent KD in advance.

## Data Availability

The datasets used and/or analyzed during the current study are available from the corresponding author on reasonable request.

## Abbreviations

KD: Keshan disease
ECG: Electrocardiogram
UCG: Ultrasonic cardiogram
CKD: Chronic Keshan disease
TMAO: Trimethyl-n-oxide
SCFA: Short-chain fatty acid
CRC: Cardiovascular research center
LKD: Latent Keshan disease
LOCF: Last-observation-carried-forward
IPAQ: Physical activity questionnaire
CBC: Hematological parameters
HDL: High-density lipoprotein cholesterol
LDL: Low-density lipoprotein cholesterol
